# Comparative responsiveness of outcome measures for total knee arthroplasty

**DOI:** 10.1016/j.joca.2013.11.001

**Published:** 2014-02

**Authors:** K. Giesinger, D.F. Hamilton, B. Jost, B. Holzner, J.M. Giesinger

**Affiliations:** † Department of Orthopaedic Surgery, Kantonsspital St. Gallen, Rorschacherstrasse 95, CH-9000 St. Gallen, Switzerland; ‡ Department of Orthopaedic Surgery, University of Edinburgh, 49 Little France Crescent, Edinburgh EH16 4SA, UK; § Department of Psychiatry and Psychotherapy, Innsbruck Medical University, Anichstr. 35, A-6020 Innsbruck, Austria; ‖ Department of Psychosocial Research and Epidemiology, The Netherlands Cancer Institute, Plesmanlaan 121, 1066 CX Amsterdam, The Netherlands

**Keywords:** Responsiveness, Patient-reported outcome, Forgotten joint score, WOMAC score, Knee arthroplasty

## Abstract

**Objective:**

The aim of this study was to compare the responsiveness of various patient-reported outcome measures (PROMs) and clinician-reported outcomes following total knee arthroplasty (TKA) over a 2-year period.

**Methods:**

Data were collected in a prospective cohort study of primary TKA. Patients who had completed Forgotten Joint Score-12 (FJS-12), Western Ontario and McMaster Universities (WOMAC) osteoarthritis (OA) index, EQ-5D, Knee Society Score and range of movement (ROM) assessment were included. Five time points were assessed: pre-operative, 2 months, 6 months, 1 year and 2 years post-operative.

**Results:**

Data from 98 TKAs were available for analysis. Largest effect sizes (ES) for change from pre-operative to 2-month follow-up were observed for the Knee Society Score (KSS) Knee score (1.70) and WOMAC Total (−1.50). For the period from 6 months to 1 year the largest ES for change were shown by the FJS-12 (0.99) and the KSS Function Score (0.88). The EQ-5D showed the strongest ceiling effect at 1-year follow-up with 84.4% of patients scoring the maximum score. ES for the time from 1- to 2-year follow-up were largest for the FJS-12 (0.50). All other outcome measures showed ES equal or below 0.30.

**Conclusion:**

Outcome measures differ considerably in responsiveness, especially beyond one year post-operatively. Joint-specific outcome measures are more responsive than clinician-reported or generic health outcome tools. The FJS-12 was the most responsive of the tools assessed; suggesting that joint awareness may be a more discerning measure of patient outcome than traditional PROMs.

## Introduction

The outcomes of total knee arthroplasty (TKA) can be assessed with various methods; implant survivorship, image-based assessment, clinical assessment and patient-reported outcome measures (PROMs). While the first three modalities are objective in nature, patient report can provide a subjective measure of the patients' perception of the success of an intervention.

The importance of including patients' views on treatment outcome in orthopaedics has been well established in recent years and a variety of patient-reported measures are available^1^. Furthermore self-reported questionnaires are a potentially cost-effective way of monitoring patient outcome in large volumes. PROMs can be broadly dichotomised into generic health status questionnaires such as the EQ-5D or SF-36 (that assess the individuals overall quality of life) and disease/joint-specific tools such as the Western Ontario and McMaster Universities (WOMAC) score which focus on specific constructs such as pain, stiffness and joint function in activities of daily living^2^. These latter examples allow a more focused evaluation of an intervention such as TKA. The most common orthopaedic patient-reported outcome (PRO) tools have been extensively analysed regarding their validity and reproducibility^3^, ^4^, ^5^. More recently researchers have turned to assess the responsiveness and floor/ceiling effects^6^, ^7^. Responsiveness to change is of particular importance in longitudinal studies where the scoring should reflect changes over time. If a questionnaire is not sufficiently responsive to the construct being assessed, it will not capture changes at follow-up, which is especially important in mid-to-long-term studies where changes in the patients' pain and function are typically not as pronounced as in the early post-operative phase. This is of direct relevance to measuring PRO following TKA where patient function changes markedly in the early post-operative phase but is followed by more subtle changes over time^8^.

Previous studies of instrument responsiveness however tended to focus on comparison of general health measures vs joint-specific measures^6^, ^9^ or covered follow-up only up to 12 months^10^, ^11^, ^12^, ^13^. Comprehensive analyses of multiple outcome assessment tools at various time points over 2 years are lacking.

The aim of this study is to compare the responsiveness of various PROMs (FJS-12, WOMAC score, EQ-5D) and clinician-reported outcomes (Knee Society Score, range of motion) following TKA.

## Patients and methods

### Sample population

Data were collected in a prospective cohort study of primary TKA between 2007 and 2009 at Kantonsspital St. Gallen, Switzerland. This was a pragmatic study that reflected local surgical practice at the time using both, mobile and fixed bearing designs. Informed consent was obtained from the participants and ethical approval was granted by the local ethics committee. Patients who had completed FJS-12, WOMAC score, EQ-5D and Knee Society Score (KSS) were included. Participants were assessed at five different time points: pre-operatively, and at 2, 6, 12 and 24 months post-operatively. Socio-demographic and clinical data included gender, Body Mass Index (BMI), age at time of surgery and side of implant.

### Outcome measures

A single experienced study performed the clinical examinations and handed over the questionnaires to the patients who completed them independently.

#### WOMAC

WOMAC osteoarthritis (OA) index is a widely used self-report outcome measure in patients with lower limb OA that was introduced by Bellamy and Buchanan^14^. The original score with 5-point Likert response categories consists of 24 questions covering three dimensions: pain (five questions), stiffness (two questions), and function (17 questions). The WOMAC has been extensively tested for validity, reliability, feasibility, and responsiveness for measuring changes after different OA interventions^14^, ^15^, ^16^, ^17^ and has also been evaluated in an electronic form^18^. WOMAC scores were linearly transformed to a 0–100 scale with higher scores indicating more severe impairment.

#### Forgotten Joint Score (FJS-12)

The FJS-12 is a recently published PRO scale to assess joint awareness in hips and knees during various activities of daily living^19^, ^20^. It uses a 5-point Likert response format, consisting of 12 equally weighted questions with the raw score transformed to range from 0 to 100 points. High scores indicate good outcome, i.e., a high degree of being able to forget about the affected joint in daily life. In its validation study^19^ it showed a low ceiling effect and high internal consistency (Cronbach's Alpha 0.95) and discriminated well between patient groups known to show different outcome.

#### EQ-5D

The EQ-5D is a standardised generic quality of life assessment instrument with five items for use as a measure of self-reported general health^21^. Applicable to a wide range of health conditions and treatments, it provides a simple descriptive profile and a single index value for health status. It is one of the internationally most frequently used measures to gain quality of life scores for analysis in health economics as utility weights (ranging from 0 to 1) for calculating quality of life adjusted life years (QALYs) can be obtained^22^.

#### KSS

The KSS^23^ is a widely used clinician-reported outcome score with good published validity data^24^. The clinical part (Knee Score) of the KSS covers pain, range of movement (ROM), alignment and stability. The functional part (Function Score) of the KSS covers the patient's mobility (walking distance and stairs) and potential walking aids. Score range of the KSS is from 0 to 100 points for each part with higher scores indicating less severe impairment.

#### ROM

Active measures of flexion and extension were determined using universal goniometry. A high level of accuracy has been previously demonstrated assessing knee range of motion with this instrument in the clinical setting^25^ and specifically in this patient group^26^. All measurements were made by the study nurse.

### Statistical analysis

Sample characteristics are given as means, standard deviations (SDs), ranges, and frequencies. As measures of responsiveness we provide effect sizes (ES, mean difference divided by SD at earlier assessment), standardised response means (SRMs, mean change divided by the standard deviation of the change score) and relative validity (RV). RV was obtained from the ratio of the F-statistics from an analysis of variance for repeated measures, comparing two time points. As a reference measure (the denominator) we used the WOMAC total scale for all time points. In addition, we provide percentages of patients obtaining the highest or the lowest possible score on a measure (i.e., floor and ceiling effects). Statistical analyses were performed with SPSS 20.0.

## Results

### Patient characteristics

During the study period 537 patients underwent TKA at our institution. Our part-time study nurse recruited 98 of these for the study. Mean age at baseline was 68.1 years (SD 8.6), 49% were female (Table I). The number of subjects for whom data was available varied according to the different time points as shown in Table II. All available data points were included in the analyses.

**Table I:** Patient characteristics at baseline (pre-operatively, *n* = 98)

Age	Mean (SD)	68.1 (8.6)	
	Range	49–80	
Sex	Men	51.0%	*N* = 50
	Women	49.0%	*N* = 48
Side	Left	49.0%	*N* = 48
	Right	51.0%	*N* = 50
BMI	Mean (SD)	28.8 (4.5)	
	Range	19–41

**Table II:** Descriptive statistics of outcome measures

	Pre-surgery			2 Months			6 Months			1 Year			2 Years		
	Mean	SD	*N*	Mean	SD	*N*	Mean	SD	*N*	Mean	SD	*N*	Mean	SD	*N*
EQ-5D (UK)	0.56	0.28	96	0.81	0.23	93	0.89	0.22	95	0.94	0.19	96	0.94	0.17	91
FJS-12∗	–	–		20.9	17.4	87	41.7	25.9	90	67.3	27.2	94	80.8	25.8	91
WOMAC pain	47.4	16.0	91	23.1	13.6	92	13.7	10.0	91	6.8	8.7	96	4.6	9.1	91
WOMAC stiffness	49.3	30.3	88	24.9	22.0	92	12.2	14.2	91	7.8	12.6	96	4.4	11.1	91
WOMAC function	49.5	17.0	90	27.0	14.7	92	16.0	11.9	91	6.9	9.1	96	4.3	8.0	91
WOMAC total	48.4	14.9	87	26.0	13.8	92	15.2	10.5	91	6.9	8.6	96	4.4	8.0	91
ROM	112.2	22.2	97	107.9	11.8	97	114.6	15.1	95	120.4	9.8	96	120.8	9.8	90
KSS knee score	49.4	17.6	77	79.3	10.5	78	84.8	10.2	86	91.7	8.3	88	93.7	9.2	85
KSS function score	67.4	15.6	77	57.4	16.5	78	77.2	13.9	86	89.5	13.6	88	88.9	13.6	85

∗ Not administered pre-operatively.

### Responsiveness over time

To highlight how the different measures perform over different time-intervals following surgery we analysed data by investigating responsiveness compared to baseline and also to the previous follow-up assessment. Presenting responsiveness indices this way allows to demonstrate more clearly at which time point after surgery the various measures are able to capture change. Baseline comparisons are also detailed in the Table III, Table IV.

**Table III:** ES of outcome measures

	Pre-surgery – 2 months		Pre-surgery – 6 months		Pre-surgery – 1 year		Pre-surgery – 2 years		2 Months–6 months		6 months–1 year		1 Year–2 years	
	*N*	ES	*N*	ES	*N*	ES	*N*	ES	*N*	ES	*N*	ES	*N*	ES
EQ-5D (UK)	93	0.87	95	1.18	96	1.36	91	1.36	93	0.37	95	0.21	91	0.02
FJS-12∗	–	–	–	–	–	–	–	–	87	1.20	90	0.99	91	0.50
WOMAC pain	91	−1.52	91	−2.11	91	−2.54	91	−2.68	91	−0.69	91	−0.69	91	−0.25
WOMAC stiffness	88	−0.81	88	−1.22	88	−1.37	88	−1.48	91	−0.57	91	−0.31	91	−0.27
WOMAC function	90	−1.32	90	−1.97	90	−2.51	90	−2.66	91	−0.75	91	−0.76	91	−0.29
WOMAC total	87	−1.50	87	−2.23	87	−2.79	87	−2.95	91	−0.79	91	−0.78	91	−0.30
ROM	97	−0.19	95	0.11	96	0.37	90	0.39	95	0.56	95	0.38	90	0.04
KSS knee score	77	1.70	77	2.01	77	2.40	77	2.52	78	0.52	86	0.68	85	0.24
KSS function score	77	−0.64	77	0.63	77	1.42	77	1.38	78	1.20	86	0.88	85	−0.04

∗ Not administered pre-operatively.

**Table IV:** SRM and RV of outcome measures

	Pre-surgery – 2 months		Pre-surgery – 6 months		Pre-surgery – 1 year		Pre-surgery – 2 years		2 Months–6 months		6 Months–1 year		1 Year–2 years	
	SRM	RV	SRM	RV	SRM	RV	SRM	RV	SRM	RV	SRM	RV	SRM	RV
EQ-5D (UK)	0.69	0.32	1.16	0.33	1.13	0.20	1.11	0.22	0.35	0.34	0.19	0.03	−0.14	0.07
FJS-12∗	–	–	–	–	–	–	–	–	0.84	0.94	0.99	1.01	0.30	3.27
WOMAC pain	−1.18	1.19	−2.04	0.98	−2.41	0.86	−2.33	0.93	−0.78	0.79	−0.65	0.56	−0.27	0.78
WOMAC stiffness	−0.53	0.27	−1.05	0.25	−1.24	0.22	−1.36	0.31	−0.48	0.24	−0.25	0.06	−0.26	0.80
WOMAC function	−0.91	0.83	−1.93	0.87	−2.45	0.88	−2.33	0.92	−0.81	0.90	−0.90	0.97	−0.29	0.81
WOMAC total†	−0.87	1.00	−2.10	1.00	−2.65	1.00	−2.47	1.00	−0.86	1.00	−0.90	1.00	−0.31	1.00
ROM	−0.20	0.03	0.05	<0.01	0.37	0.02	0.42	0.03	0.15	0.37	0.41	0.19	−0.25	<0.01
KSS knee score	1.53	1.02	1.70	0.54	2.15	0.55	2.40	0.78	0.52	0.26	0.84	0.57	0.17	0.25
KSS function score	−0.52	0.12	0.73	0.10	1.23	0.18	1.08	0.16	1.14	1.26	0.89	0.64	−0.09	0.08

∗ Not administered pre-operatively.

† Reference measure (denominator) for calculating RV.

### Pre-operative to 2-month follow-up

Largest ES for change from pre-operative to 2-month follow-up were observed for the KSS knee score (1.70), WOMAC-pain (−1.52) and WOMAC total (−1.50). In contrast, range of motion only changed little with an ES of −0.19. SRM was biggest for WOMAC pain (−1.18) and WOMAC function (−0.91) and smallest for ROM (−0.20). At baseline, only WOMAC stiffness showed floor and ceiling effects with 12.4% of the patients obtaining the lowest possible score and 14.6% the highest possible score. At 2-months follow-up most pronounced floor and ceiling effects were observed for the EQ-5D (39.4% highest score) and again WOMAC stiffness (29.0% lowest score). Score change of the FJS-12 could not be calculated as this score was not administered pre-operatively. Further details are given in Table II, Table III, Table IV, Table V.

**Table V:** Floor and ceiling effects of outcome measures

	Pre-surgery		2 Months		6 Months		1 Year		2 Years	
Floor/Ceiling effects∗	Floor	Ceiling	Floor	Ceiling	Floor	Ceiling	Floor	Ceiling	Floor	Ceiling
EQ-5D (UK)	0.0	0.0	0.0	39.4	0.0	67.4	0.0	84.4	0.0	81.3
FJS-12†	–	–	8.0	0.0	4.4	0.0	0.0	3.2	2.2	33.0
WOMAC pain	0.0	0.0	2.2	0.0	7.7	0.0	41.7	0.0	65.9	0.0
WOMAC stiffness	12.4	14.6	29.0	2.2	51.6	0.0	64.6	0.0	82.4	0.0
WOMAC function	0.0	0.0	1.1	0.0	1.1	0.0	20.8	0.0	49.5	0.0
WOMAC total	0.0	0.0	1.1	0.0	1.1	0.0	15.6	0.0	39.6	0.0
Range of motion‡	–	–	–	–	–	–	–	–	–	–
KSS knee score	0.0	0.0	0.0	0.0	0.0	4.7	0.0	27.3	0.0	37.6
KSS function score	0.0	3.8	0.0	1.3	0.0	14.6	0.0	53.4	0.0	47.1

∗ Percentages for lowest (floor) and highest (ceiling) possible score on a scale.

† Not administered pre-surgery.

‡ Concept of floor and ceiling effect not applicable.

### 2-Month to 6-month follow-up

From 2-month to 6-month follow-up the biggest change in terms of ES was found for the FJS-12 and the KSS function score (both 1.20). The KSS knee score (0.52) and the EQ-5D (0.37) showed the smallest change for this period. SRM was smallest for the EQ-5D (0.35) and biggest for the KSS function score (1.14) and the WOMAC total score (−0.86). At 6-month follow-up the most pronounced floor and ceiling effects were found for the EQ-5D (67.4% highest score), the WOMAC stiffness score (51.6%) and the KSS function score (14.6% highest score). Further details are given in Table II, Table III, Table IV, Table V.

### 6-Month to 1-year follow-up

For the period from 6 months to 1 year the greatest ES for change were shown by the FJS-12 (0.99) and the KSS function score (0.88). The FJS-12 was also the largest in terms of SRM (0.99), followed by the WOMAC function and total score (both −0.90) and the KSS function score (0.89). Again the EQ-5D and the WOMAC stiffness score performed worst with regard to ES and SRM. These two scores also showed the strongest floor and ceiling effects at 1-year follow-up (EQ-5D 84.4% highest score and WOMAC stiffness 64.6% lowest score). At this time point the FJS-12 was the only score that had less than 10% in the highest or lowest category. Further details are given in Table II, Table III, Table IV, Table V.

### 1–2-year follow-up

ES for the time from 1- to 2-year follow-up were biggest for the FJS-12 (0.50). All other scores showed ES equal or below 0.30. SRM was highest for the WOMAC Total score (0.31) and the FJS-12 (0.30). ROM remained constant at a mean of 120° showing no ceiling effect as TKA patients' ROM is naturally less than a healthy individual's ROM. All other outcome measures showed substantial floor and ceiling effects. The FJS-12 had 33.0% of patients showing the highest score followed by the KSS Knee score (37.6%) and the WOMAC Total score (39.6%). Further details are given in Table II, Table III, Table IV, Table V.

## Discussion

This study demonstrates that outcome measures widely used in orthopaedic research differ substantially with regard to their responsiveness. Previous authors have highlighted differences between various tools, however have focused on early outcome, typically comparing two instruments ability to assess change over 6–12 months post-operatively^10^, ^11^, ^12^, ^13^, ^27^.

Complicating outcome assessment interpretation is the fact that the various scores have differing (sometimes substantial) ceiling effects, e.g., they are not capturing change due to a lack of discriminatory power of the scores as opposed to a lack of change.

A particular strength of this study is the comprehensive assessment of various outcome tools over five time points, which allows a more detailed analysis of the behaviour of the different tools into the later recovery phase. There is scant data on PROM responsiveness for 2-year follow-up periods and longer. Whereas Browne *et al.*^28^ suggested to follow-up patients until 1 year post-operatively, our data demonstrate the need of longer follow-up periods. We captured change between 12 and 24 months using responsive measures. The need for follow-up beyond one year has been recognised and is also reflected by journal author guidelines^29^. It is of note that orthopaedic journal author guidelines started to require 2-year outcome data for clinical studies involving new implants despite the fact that the ability of various PROMs to capture change over this time frame still needs further investigation.

The sex ratio we report in this study was unexpectedly equal, however, this reflects the patient throughput in our clinics on the 2 days/week the study nurse was present to recruit. To check that this had no confounding effect on our study findings we compared weighted and unweighted ES of the measures. We weighted the study cohort to reflect the sex ratio from our local arthroplasty database (59.8% female) and calculated the difference. This did not influence the results presented in the manuscript and we can therefore be confident in the analysis presented.

The joint-specific scores (WOMAC score and the FJS-12) showed the highest responsiveness in terms of ES and SRMs compared to the KSS or the EQ-5D. The KSS and ROM measurement was able to detect change up to 1 year follow-up. However, the KSS barely improved (1.4 points) between the 1 and 2-year follow-up and ROM remained constant at 120° (Table II, Table III, Table IV, Table V). The two parts of the KSS (Knee and function score) also showed limited responsiveness in terms of ES and SRM between 1- and 2-year follow-up but performed substantially better during the first post-operative year. The decreasing function score of the KSS from pre-operatively to 2 months post-operatively is mainly due to the use of walking aids. The KSS is very sensitive to this question by subtracting 20 points if crutches are used. However, at our hospital we often recommend crutches to elderly patients for 2 months for safety reasons (especially in winter) so this may well have skewed the KSS in our study. McKay *et al.*^30^ investigated the responsiveness of PROMs and objective measures pre-operatively and 6 weeks post-operatively in knee OA patients. For the WOMAC function scale these authors reported a very similar ES (1.17) and SRM (0.90) for post-operative change at 6-week follow-up as found in our study. For objective measures ES were considerably smaller (flat surface walking test 0.38, stair ascent/descent 0.52, and quadriceps strength 0.68).

In this study, the EQ-5D performed very poorly in terms of responsiveness, which is related to the vast ceiling effect from 6-months follow-up onwards (e.g., 84.4% of the patients had the highest possible score at 1-year follow-up, Table II, Table III, Table IV, Table V). Similarly, Ko *et al.*^6^ found better responsiveness of joint-specific measures in TKA patients compared to the generic SF-36, and the clinician-reported KSS. These results highlight the importance of the disease/joint-specific PROMs for orthopaedic outcome research as they provide a valuable means to sensitively capture changes in patient's condition especially once the post-operative rehabilitation phase has been completed.

However, joint-specific measures also show different responsiveness. Theiler *et al.*^12^ compared the WOMAC with the clinician-reported Lequesne algo-functional index at baseline, 6 and 12 months in patients undergoing total hip arthroplasty (THA) and TKA and found superior responsiveness of the patient-reported WOMAC score. In a recent study Williams *et al.*^7^ compared responsiveness of the WOMAC, the Knee Outcome Survey – Activities of Daily Living Scale (ADLS) and the Lower extremity Functional Scale (LEFS) in patients with knee OA participating in a rehabilitation programme (2, 6 and 12 months after the start of the programme). In contrast to our study TKA was an exclusion criterion and patients were in a better condition (baseline WOMAC total score was 28.1 points vs pre-operative WOMAC total score was 48.4 points in our study). When comparing their baseline with 2-month follow-up ES for change were 0.33 for the ADLS, 0.32 for the LEFS and 0.43 for the WOMAC (values we have calculated from summary tables in their manuscript). This suggests that in patients with a lower symptom burden, the responsiveness of these specific outcome measures is poor. Similarly, in the later post-operative phase after TKA in our study, ES for change were low for the WOMAC score (0.30 between 1 and 2 years follow-up). The FJS-12 was more responsive with an ES of 0.50 in that time period.

Generic scores such as the EQ-5D failed to detect change after the early rehabilitation phase since 81% TKA patients report good outcomes following surgery^8^, ^31^. Therefore joint-specific PROMs are needed to capture change over time or to pick up differences between two groups in a cross-sectional study design. The FJS-12, a measure of patients' joint awareness during activities of daily living, performed best with regard to ES of changes between from 2 and 6-months, 6 and 12 months and between 1 and 2-years follow-up (Table II, Table III, Table IV, Table V). From a logistical and patient compliance point of view, it is notable that these advantageous measurement characteristics accompany a low number of questions asked.

In most of the outcome measures (WOMAC, ROM, KSS) SD decreased over time (halving between pre-op assessment and 2-year follow-up). This is very important in the interpretation of ES, as the SD is the denominator. In the early recovery phase, floor/ceiling effects are less pronounced because data are more normally distributed. This results in larger SDs (ES denominator). Thus, the same mean difference results in lower ES in the early recovery phase compared to the later phase. It is critical to consider this statistical artifact (which affects SRM in a similar manner) when interpreting the results in Table II, Table III, Table IV, Table V.

Beyond ES, RV allows for comparative analysis of individual scores. According to Fayers and Hays^32^ RV gives the ratio of sample sizes “*that would be required to detect the known group difference using one measure versus the other”*. Therefore RV allows comparison of sample size needed for each instrument. Our data highlight that the EQ-5D requires 5 times as many patients as the WOMAC score to demonstrate baseline to 1-year change. It requires 10 times the number of patients compared to the WOMAC score to capture change between 1 and 2 years post-operatively. For longer term follow-up (2 years) the FJS-12 requires only one third of the number of patients compared to the WOMAC score. These are important considerations when powering outcome studies with PROMs.

The good responsiveness to change of the FJS-12 is perhaps because this score is based on a more discerning construct. A ‘forgotten joint’ (i.e., that the patient has no awareness of the affected joint during various activities of daily living) is very hard to accomplish. The relatively large ES of this score at 1- and 2-year follow-up are beneficial with regard to powering outcome studies over a longer time span, as substantial floor and ceiling effects compromise responsiveness.

## Conclusion

Outcome measures differ considerably in responsiveness, especially beyond one year post-operatively (i.e., when comparing scores at 1- and 2-year follow-up). Joint-specific self-reported outcome measures are more responsive than clinician-reported or generic health outcome tools. The FJS-12 was the most responsive tool assessed. This suggests that joint awareness may be a more discerning measure of patient outcome than traditional PROMs.

## Author's contributions

KG and JMG conceived the study objective. All authors participated in the study design. KG coordinated data collection. JMG and KG performed the statistical analysis and interpreted the results. All authors helped to outline the manuscript. KG, JMG and DH drafted the manuscript. All authors read and approved the final version.

## Competing interests

None.

## Role of funding source

This study had no specific funding or sponsor.
